# Genetic silencing of Nrf2 enhances X-ROS in dysferlin-deficient muscle

**DOI:** 10.3389/fphys.2014.00057

**Published:** 2014-02-19

**Authors:** Ponvijay Kombairaju, Jaclyn P. Kerr, Joseph A. Roche, Stephen J. P. Pratt, Richard M. Lovering, Thomas E. Sussan, Jung-Hyun Kim, Guoli Shi, Shyam Biswal, Christopher W. Ward

**Affiliations:** ^1^Department of Environmental Health Sciences, Bloomberg School of Public Health, Johns Hopkins UniversityBaltimore, MD, USA; ^2^Department of Healthcare Sciences, Eugene Applebaum College of Pharmacy and Health Sciences, Wayne State UniversityDetroit, MI, USA; ^3^Department of Orthopaedics, University of Maryland School of MedicineBaltimore, MD, USA; ^4^Department of Organizational Systems and Adult Health, University of Maryland School of NursingBaltimore, MD, USA

**Keywords:** Nrf2, X-ROS, ROS, dysferlin, dystrophy

## Abstract

Oxidative stress is a critical disease modifier in the muscular dystrophies. Recently, we discovered a pathway by which mechanical stretch activates NADPH Oxidase 2 (Nox2) dependent ROS generation (X-ROS). Our work in dystrophic skeletal muscle revealed that X-ROS is excessive in dystrophin-deficient (*mdx*) skeletal muscle and contributes to muscle injury susceptibility, a hallmark of the dystrophic process. We also observed widespread alterations in the expression of genes associated with the X-ROS pathway and redox homeostasis in muscles from both Duchenne muscular dystrophy patients and *mdx* mice. As nuclear factor erythroid 2-related factor 2 (Nrf2) plays an essential role in the transcriptional regulation of genes involved in redox homeostasis, we hypothesized that Nrf2 deficiency may contribute to enhanced X-ROS signaling by reducing redox buffering. To directly test the effect of diminished Nrf2 activity, Nrf2 was genetically silenced in the A/J model of dysferlinopathy—a model with a mild histopathologic and functional phenotype. Nrf2-deficient A/J mice exhibited significant muscle-specific functional deficits, histopathologic abnormalities, and dramatically enhanced X-ROS compared to control A/J and WT mice, both with functional Nrf2. Having identified that reduced Nrf2 activity is a negative disease modifier, we propose that strategies targeting Nrf2 activation may address the generalized reduction in redox homeostasis to halt or slow dystrophic progression.

## Introduction

In dystrophin-deficient heart and skeletal muscle, we recently identified that NADPH oxidase 2 (Nox2) dependent reactive oxygen species (ROS) production (i.e., X-ROS) is dramatically enhanced with stretch or contraction and contributes to the enhanced susceptibility to injury (Prosser et al., 2011; Khairallah et al., 2012). Furthermore, we demonstrated that this enhanced X-ROS is temporally progressive as X-ROS is virtually non-existent in young (~8 week) and in great excess in mature adult (~7 month) dystrophic animals.

Limb girdle muscular dystrophy type 2B (LGMD2B), Miyoshi myopathy, and distal myopathy are progressive, late-onset dystrophies linked to mutations in, or loss of, the dysferlin protein that present clinically as weakness in the proximal or distal muscles with elevated serum creatine kinase (CK) and muscle degeneration (Nigro, 2003; Klinge et al., 2008; Amato and Brown, 2011). We reported that aged (~12 month) dysferlin-deficient murine muscle also exhibits amplified X-ROS signaling (Prosser et al., 2013), a finding congruent with the recent reports suggesting that oxidative stress underscores the late onset and temporal progression of dysferlinopathy (Terrill et al., 2013). Despite these findings, it is unclear what manifests the age-dependent increase in X-ROS in these dystrophic models.

Within the healthy muscle cell, ROS are generated as both a by-product of metabolism and as effectors of signaling cascades. Excess ROS is counteracted by proteins that promote reduction-oxidation (i.e., redox) chemistry under normal conditions. Our recent work identified a down-regulation in the transcriptional profile of redox related genes in murine adult dystrophin-deficient (*mdx*) *tibialis anterior* muscle (Khairallah et al., 2012), suggesting that a decrease in redox related proteins may induce a shift to a more oxidized environment and drive dysfunction in dystrophic muscle.

Nuclear factor erythroid 2-related factor 2 (Nrf2) is a transcription factor ubiquitously expressed in most tissues, but most abundantly in kidney, striated muscle, and lung. Nrf2 is bound to Kelch like-ECH-associated protein 1 (Keap1) in the cytosol. In response to oxidative stress, the Nrf2–Keap1 interaction is disrupted and Nrf2 translocates into the nucleus where it activates the Antioxidant Response Element (ARE) which transcriptionally activates many antioxidative genes. In this role, Nrf2 activity has been demonstrated to be an important disease modifier in many oxidative/inflammatory diseases, such as asthma, sepsis, cerebral hemorrhage, and pulmonary fibrosis, where decreased Nrf2 activity exacerbates disease progression (Cho et al., 2004; Rangasamy et al., 2005; Thimmulappa et al., 2006; Wang et al., 2007).

As we recently identified amplified X-ROS signaling in aged dysferlin-deficient A/J mice (Prosser et al., 2013) and others have now reported a temporal progression in muscle oxidation in dysferlin-deficient muscle (Terrill et al., 2013), we sought to directly test the hypothesis that a decrease in Nrf2 activity could serve as a disease modifier in dystrophic muscle. To this end we genetically silenced Nrf2 in the A/J mouse model of dysferlinopathy. We chose this model as it is considered a relatively weak model of dysferlinopathy due to its lack of significant functional deficits and mild, late onset, histological presentation (Von der Hagen et al., 2005; Kobayashi et al., 2010; Gayathri et al., 2011; Grounds and Shavlakadze, 2011; Kobayashi et al., 2011; Terrill et al., 2013). Examination of A/J-Nrf2^−/−^ muscle at 12 months of age revealed a dramatic enhancement in X-ROS, significant decreases in muscle function, and histological evidence of myopathy. Taken together, these results support decreased Nrf2 activity as a disease modifier in dysferlinopathy.

## Materials and methods

### Mice

Dysferlin-null A/J mice were obtained from Jackson Laboratories and bred in our facility. A mouse model of dysferlinopathy with ablated Nrf2 (A/J-Nrf2^−/−^) was obtained by backcrossing Nrf2^−/−^ mice (C57BL/6J background) 7 times with A/J mice. All experimental protocols conducted on the mice were performed in accordance with NIH guidelines and were approved by the Johns Hopkins University and University of Maryland, Baltimore Animal Care and Use Committees.

### Isolation of flexor digitoris brevis (FDB) myofibers

After euthanasia by CO_2_ inhalation, the FDB muscle was harvested bilaterally and incubated in Dulbecco's Modified Eagle's Medium (DMEM; Life Technologies, Grand Island, NY, USA) with 1 μ l/ml gentamicin and 4 mg/ml collagenase A (Roche Applied Science, Indianapolis, IN, USA) for up to 3 h as previously described (Ziman et al., 2010; Dorsey et al., 2012). Single intact myofibers were then isolated by gentle trituration.

### Myofiber attachment and stretch

All experiments were performed as described (Khairallah et al., 2012). In brief, we used a custom rotating glass-bottom chamber (Four-hour Day Foundation, Towson, MD, USA) equipped with bath perfusion and mounted on Zeiss Radiance fluorescent laser scanning confocal attached to an Olympus IX-70 inverted microscope (Olympus Corp., Centre Valley, PA, USA). A high-sensitivity force transducer (KG7) and length controller (World Precision Instruments, Sarasota, FL, USA) were mounted on folded motorized micromanipulators (Siskiyou, Grants Pass, OR, USA). A single FDB fiber was attached to both the force transducer and the length controller using micro-tweezers coated with the biological adhesive, MyoTak (Ionoptix, Milton, MA, USA). Using a high-speed video sarcomere length system (HSVL; Aurora Scientific, Aurora, Ontario, Canada), initial sarcomere length and sarcomere length following passive stretch were monitored. To induce X-ROS, FDB fibers were challenged with a brief 5 s passive stretch (10% sarcomere length).

### Reactive oxygen species (ROS) measurements

ROS was measured as previously described (Prosser et al., 2011) using 6-carboxy-2′,7′-dichlorodihydrofluorescein diacetate (DCFH-DA, or DCF) (Invitrogen, Carlsbad, CA, USA) in dimethyl sulfoxide (DMSO). FDB fibers were suspended in HEPES-buffered Ringer's solution containing (in mM): 140 NaCl, 4 KCl, 1 MgSO_4_, 5 NaHCO_3_, 10 glucose, 10 HEPES at pH 7.3 and incubated with Ringer's solution containing DCFH-DA (10 μ M) for 30 min at room temperature (22°C). DCFH-DA-loaded cells were imaged using confocal line-scanning microscopy at 2 ms/line with the lowest laser power possible that reported a stretch dependent ROS signal. DCFH-DA fluorescence signals were processed as previously described (Prosser et al., 2011).

### Western blotting

Western blot analysis was performed as previously described (Dorsey et al., 2012). Briefly, 20 μ g of clarified muscle extract was subjected to SDS-PAGE, transferred to nitrocellulose membranes and blocked in a 5% milk solution in PBS for 1 h. The membrane was probed overnight with primary antibody at room temperature. The primary antibodies were anti-α-tubulin (DM1A, 1:1000; Sigma-Aldrich), anti-β-tubulin (AA12.1, 1:1000, Developmental Studies Hybridoma Bank), anti-Glu-tubulin (1:1000, Millipore, Temecula, CA, USA), anti-gp91^phox^ (1:1000, BD Transduction Laboratories, Lexington, KY, USA), and anti-GAPDH (1:4000, Millipore). Membranes were washed twice for 10 min in 5% milk solution at room temperature, then incubated with appropriate secondary antibody (1:10,000) for 1 h at room temperature and washed in a 0.5% Tween solution in PBS twice for 10 min. Immunoblots were developed using SuperSignal PicoWest chemiluminescence (Pierce, Rockford, IL, USA) and were imaged and quantified with a SYNGENE GBox imaging system (GeneTools software). GAPDH served as a loading control.

### *In vivo* assessment of quadriceps force

Functional assessment of the quadriceps muscle was performed *in vivo* as described (Pratt et al., 2012). Mice were deeply anesthetized with isoflurane and placed in a supine position on the apparatus. The thigh was stabilized and the ankle secured onto a lever arm with the rotational axis of the knee aligned with the axis of the stepper motor/torque sensor. The femoral nerve was stimulated via subcutaneous needle determined by a series of isometric twitches and by observing isolated knee extension in the anesthetized animal. Maximal torque was assessed as the mean of 3 successive (1 min apart) maximal tetanic contractions (1 s train, 0.2 μs sq pulses, 100 Hz).

### *In vitro* muscle contractility

Muscle performance of the extensor digitorum longus (EDL) was assessed *in vitro* using methods described previously (Williams et al., 1998). In brief, single EDL muscles were surgically excised with ligatures at each tendon (5-0 silk suture) and mounted in an *in vitro* bath between a fixed post and force transducer (Aurora 300B-LR) operated in isometric mode. The muscle was maintained in physiological saline solution (PSS; pH 7.6) containing (in mM) 119 NaCl, 5 KCl, 1 MgSO_4_, 5 NaHCO_3_, 1.25 CaCl_2_, 1 KH_2_PO_4_, 10 HEPES, 10 dextrose, and maintained at 30°C under aeration with 95 O_2_/5 CO_2_ (%) throughout the experiment. Resting tension and stimulation current were iteratively adjusted for each muscle to obtain optimal twitch force. During a 5 min equilibration, single twitches were elicited at every 30 s with electrical pulses (0.2 ms) via platinum electrodes running parallel to the muscle. Optimal resting tension was determined and isometric tension was evaluated by 250 ms trains of pulses delivered at 1, 10, 20, 40, 60, 80, 100, 150 Hz. After the experimental protocol, the muscle rested for 5 min at which time muscle length was determined with a digital micrometer, muscle was trimmed proximal to the suture connections, blotted and weighed. The cross-sectional area for each muscle was determined by dividing the mass of the muscle (g) by the product of its length (*L_o_*, mm) and the density of muscle [1.06 g/cm^3^; (Mendez and Keys, 1960)] and was expressed in square centimeters. Muscle output was then expressed as isometric tension (g/cm^2^) determined by dividing the tension (g) by the muscle cross-sectional area. Maximal torque was assessed as the mean of 3 successive (1 min apart) maximal tetanic contractions (1 s train, 0.2 μs sq pulses, 100 Hz).

### Histopathology

Quadriceps muscles were fixed in 10% formalin and flash frozen. Cross-sections (5 μm) were obtained from the mid-belly of the muscle. H&E staining was performed to qualitatively assess inflammatory cell infiltrate and centrally nucleated fibers (CNFs). Picrosirius red staining was used to assess collagen and fibrosis. Digital images (20× magnification) were obtained and the area of rectus femoris was calculated using ImageJ (National Institutes of Health, Bethesda, MD). CNFs were quantified from images (100× magnification) taken at the lesion in the rectus femoris from each genotype. ImageJ was used to calculate the total area of fat accumulation (vacuolated area).

### Immunohistochemistry of oxidized phospholipids

Muscle sections of 5 μm thickness were generated from formalin fixed quadriceps were stained with antibodies generated against oxidized phospholipids (oxPL) to assess oxPL and oxidative stress. Staining was done according to the manufacturer's protocol (Dako). Digital images (20× magnification) were at the same location in the muscle from each genotype.

### Statistics

Statistical comparisons were performed using Student's *t*-test or One-Way ANOVA (SigmaStat v3.1) with significance set *a priori* to 0.05.

## Results

### Stretch-induced ROS generation is exaggerated in the Nrf2-silenced dysferlin deficient muscle

We recently reported on X-ROS, a signaling pathway by which mechanical stretch elicits Nox2 ROS signaling in heart and skeletal muscle (Prosser et al., 2011; Khairallah et al., 2012). The microtubule (MT) cytoskeleton is the mechano-transduction element that transmits the strain of stretch or contraction to Nox2, and hence, is critical for the generation of X-ROS. We further identified excess X-ROS magnitude and enhanced X-ROS protein expression (i.e., MT and Nox2 protein subunits) in dystrophin-deficient heart and skeletal muscle. In each tissue, the increased magnitude of X-ROS contributed to dysfunctional ROS, alterations in calcium signaling, and injury susceptibility. Integral to this current work, we most recently identified enhanced X-ROS and increased X-ROS-related protein expression in the A/J model of dysferlinopathy (Prosser et al., 2013).

**Figure 1:**
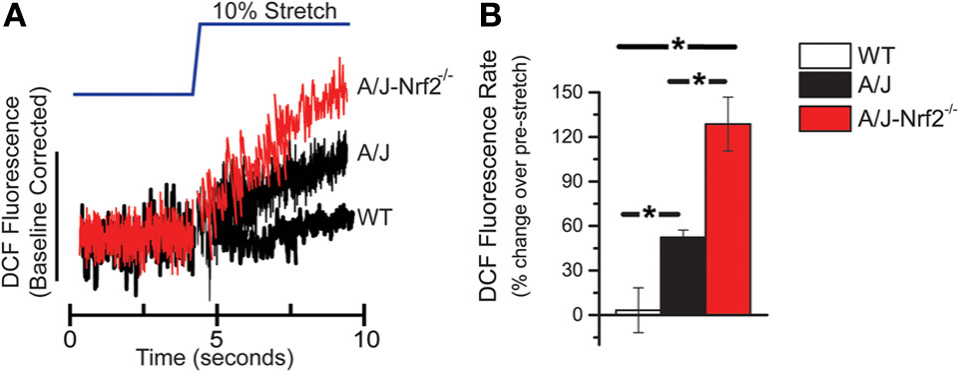
**X-ROS is enhanced in Nrf2 silenced dysferlin deficient muscle fibers**. FDB fibers from WT (*n* = 3_animals_, 8_fibers_), dysferlin deficient A/J (*n* = 3_animals_, 9_fibers_), and Nrf2-silenced A/J (*n* = 3_animals_, 11_fibers_) were loaded with the fluorescent ROS probe DCF and attached with MyoTak between a high-sensitivity force transducer and length controller. The fibers were challenged with a brief 5 s passive stretch (~10% sarcomere length) and DCF fluorescence signals **(A)** were processed as previously described (Prosser et al., 2011). The DCF fluorescent rate, expressed as a % over the pre-stretch condition, revealed that WT stretch was not different from the pre-stretch condition, dysferlin deficient A/J was significantly elevated over WT, and Nrf2 silenced A/J were elevated compared to each other genotype. The population averages are represented in the bar graph in **(B)**. ^*^p < 0.05.

Here we used an identical approach to assess the rate of ROS production in single isolated FDB myofibers from A/J-Nrf2^−/−^ mice loaded with DCF and challenged with an acute stretch of a 10% change in sarcomere length. In FDBs from 12-month dysferlin-deficient A/J mice, we confirmed a significant increase in X-ROS compared to WT controls. We found that in dysferlin-deficient FDBs with genetically silenced Nrf2, the X-ROS rate was significantly elevated above that seen in the dysferlin-deficient A/J (Figures 1A,B). This result suggests that a decrease in Nrf2 activity accelerated the temporal manifestation of X-ROS in the dysferlin-deficient A/J model.

**Figure 2:**
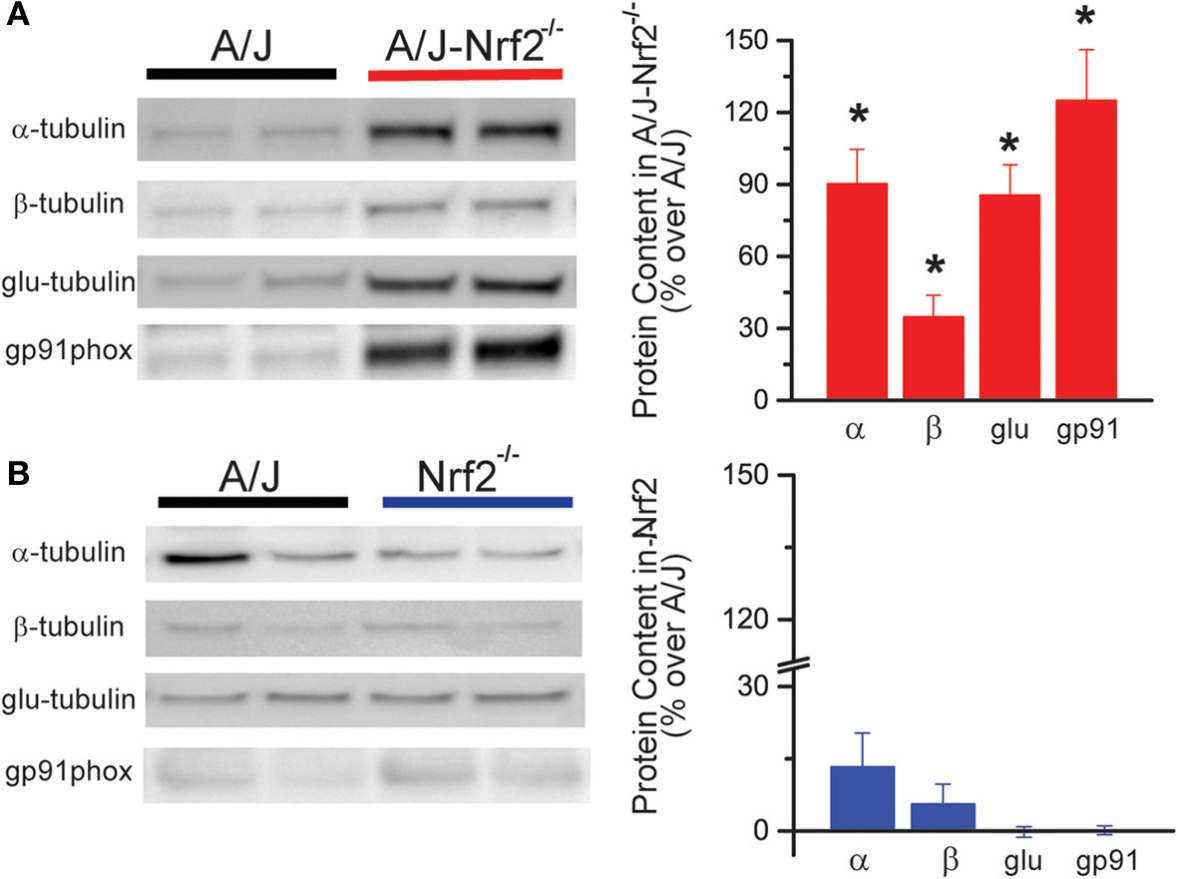
**X-ROS protein expression is elevated in Nrf2 silenced dysferlin deficient muscle**. Western blot analysis of gastrocnemius muscle from 1 year old mice identified that X-ROS protein content is significantly elevated in Nrf2 silenced A/J muscle **(A)** (*n* = 5) but not in Nrf2 silenced muscle **(B)** (*n* = 5). Percent changes in protein expression are demonstrated in the bar graphs to the right of both **(A,B)**. ^*^p < 0.05.

Western blot analysis revealed that Nrf2 silencing in the A/J resulted in the increased expression of X-ROS-related proteins, including α- and β-tubulin, glu-tubulin, and gp91^phox^ (Figure 2A), consistent with the increased magnitude of X-ROS we demonstrated in this model. Importantly, the silencing of Nrf2 alone (i.e., Nrf2^−/−^) did not result in an increased expression of these proteins, indicating that Nrf2 silencing in the dystrophic (A/J-Nrf2^−/−^) model drove this response (Figure 2B).

### Muscle function and quality is decreased in the Nrf2-silenced dysferlin deficient mouse

**Figure 3:**
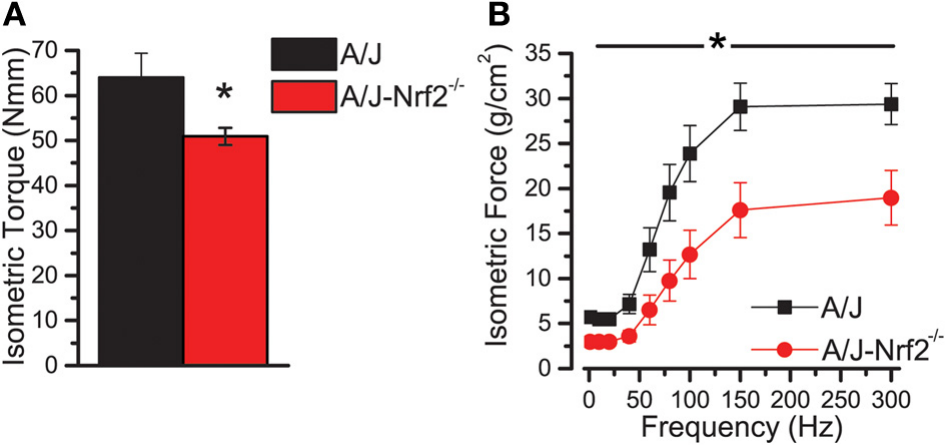
**Nrf2 silenced A/J muscle exhibits a loss-of-function phenotype. (A)** *In vivo* assessments of isomeric quadriceps torque and **(B)** *in vitro* assessments of EDL contractility via the force vs. stimulation frequency relationship both revealed significant loss-of-function phenotypes in the Nrf2 silenced dysferlin deficit A/J muscle. ^*^p < 0.05.

**Figure 4:**
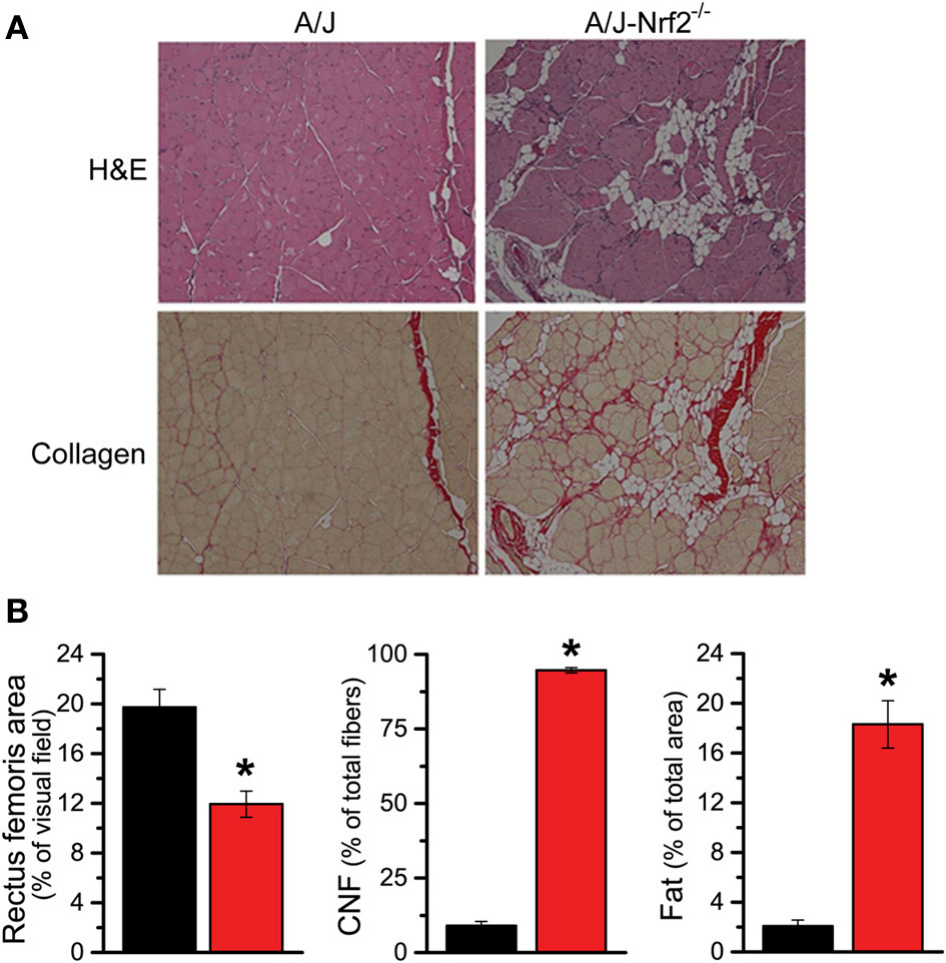
**Histological evidence of enhanced pathology in Nrf2 silenced A/J muscle. (A)** Cryosections were H&E stained to evaluate myofiber morphology and immune cell infiltrate (top) and Picrosirius red stained for assaying collagen content (bottom). **(B)** The Nrf2 silenced A/J muscle exhibited a decrease in total myofiber area, an increase in collagen content, the number of CNFs, and fatty deposits. ^*^p < 0.05.

**Figure 5:**
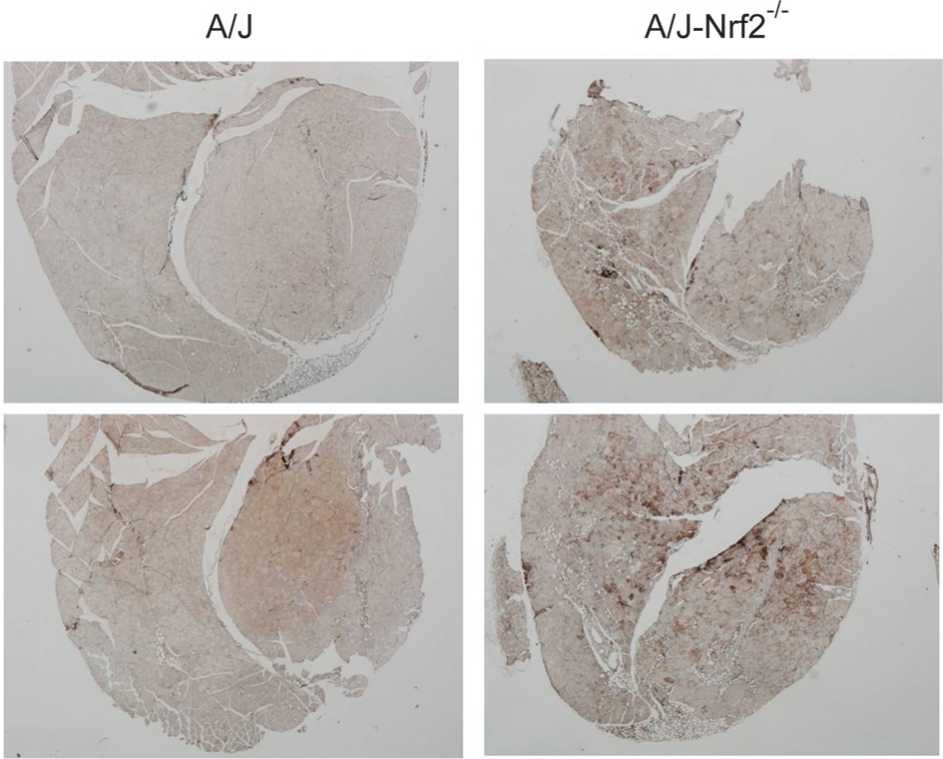
**Increased oxidative stress in of Nrf2 silenced A/J quadriceps muscle**. Quadriceps muscles (*n* = 6) were isolated from A/J and A/J-Nrf2^‒/‒^ mice at 1 year of age, and were stained with antibodies against oxPL. Sections from A/J-Nrf2^‒/‒^ muscles (right panels) exhibit excess oxPL (dark brown staining), compared to sections derived from A/J mice (left panels).

We have proposed that excessive X-ROS signaling is a proximal mechanism for the muscle damage and force deficits in dystrophic muscle. To assess the effect of Nrf2 silencing on the dysferlin-deficient A/J, we first evaluated nerve-evoked muscle function *in vivo*. Evaluation of quadriceps muscle function revealed a significant deficit in maximal isometric torque in the A/J-Nrf2^−/−^ vs. the A/J (Figure 3A) despite no difference in bodyweight (27.2 ± 2.1 vs. 28.4 ± 1.9 g). To assess muscle specific force production, we analyzed electrically evoked contractions in single EDL muscles *in vitro*. An examination of the force (normalized to the muscle cross-sectional area) vs. stimulation frequency relationship revealed a significant deficit in force producing capacity in the A/J-Nrf2^−/−^ (Figure 3B). Histological analysis of the quadriceps muscles provided insight for the deficits in muscle function following Nrf2 ablation, as A/J-Nrf2^−/−^ mice exhibit enhanced degeneration compared to A/J mice (Figure 4). Furthermore, these muscles present with increased immune cell infiltrate (Figure 4A; top, right), fibrosis (Figure 4A; bottom, right), steatosis (fat accumulation), and a concomitant decrease in total myofiber area in cross-section (Figure 4B). Taken together with the increase in the occurrence of CNFs and evidence of increased oxidative stress (Figure 5), we conclude that the A/J-Nrf2^−/−^ mouse presents with a significantly enhanced dystrophic phenotype compared to age-matched A/J mice.

## Discussion

LGMD2B, Miyoshi myopathy, and distal myopathy are progressive dystrophies that present clinically as weakness in the proximal or distal muscles with elevated serum CK and muscle degeneration (Nigro, 2003; Klinge et al., 2008; Amato and Brown, 2011). These diseases are established as late-adult onset both clinically (Gordon et al., 1993; Klinge et al., 2008; Angelini et al., 2010, 2011; Gayathri et al., 2011) as well as in preclinical models (Turk et al., 2006; Nemoto et al., 2007; Ng et al., 2012). While these disorders have been linked to the mutation or ablation of the dysferlin gene (*DYSF*) (Illarioshkin et al., 2000; Vainzof et al., 2001; Nguyen et al., 2005; Cacciottolo et al., 2011), a consensus on the mechanistic basis of disease has not been reached.

Early functional studies in murine muscle (Bansal et al., 2003; Bansal and Campbell, 2004) implicated dysferlin as an integral component for rapid repair of the sarcolemma. However, more recent reports indicate that dysferlin is also involved in vesicle fusion, cellular adhesion, MT regulation and the stabilization of sarcolemmal/transverse-tubule Ca^2+^ homeostasis (Lennon et al., 2003; Covian-Nares et al., 2010; Sharma et al., 2010; Di Fulvio et al., 2011; De Morree et al., 2013; Kerr et al., 2013). Most recently, evidence points to an age-dependent enhancement in oxidative stress in dysferlin-deficient muscle (Prosser et al., 2013; Terrill et al., 2013) leading us to speculate that a progressive enhancement in oxidative stress may temporally associate with the late onset of muscle pathology seen in dysferlinopathy.

We previously identified a decrease in redox related genes in Dmd muscle (Khairallah et al., 2012), and have identified similar profiles from interrogating publically available microarray data from clinical biopsy samples of dysferlinopathic individuals (GEO repository; data not shown). Together, the increased lipid peroxidation in muscles of dysferlinopathy patients (Renjini et al., 2012), the progressive oxidation of muscle of dysferlin deficient A/J mice (Terrill et al., 2013), and the increase in X-ROS in dysferlin-deficient muscle (Prosser et al., 2013) all *indirectly* support a decline in Nrf2 activity as a factor in the onset and pathogenic progression of the disease. In further support of Nrf2's role, a decline in Nrf2 activity, increased oxidative stress and reduced antioxidant defense seem to be general phenomena in chronic diseases and aging (Malhotra et al., 2008; Safdar et al., 2010; Sykiotis and Bohmann, 2010), therefore it will be important to also evaluate the effect of Nrf2 silencing in the context of aging WT muscle, a comparison not made in this study.

Our sentinel measure of stretch activated X-ROS was previously shown to be elevated in the dysferlin-deficient A/J mouse, and here we demonstrate a dramatic (>2-fold) elevation in X-ROS in A/J-Nrf2^−/−^ muscle compared to the A/J (*c.f*. Figure 1). As discussed above, ROS production is countered by the endogenous cellular redox buffering systems such that a detectable increase in ROS production could be due to an increase in ROS production via more ROS producing sources (i.e., Nox2 or mitochondrial metabolism), a decrease in redox buffering, or a combination of both. Interestingly, the silencing of Nrf2 in the A/J resulted in an increase in X-ROS proteins (α-, β-tubulin and gp91^phox^), which may suggest a more complex interplay of redox homeostasis and the transcriptional regulation of these X-ROS-related proteins. Presently, pharmacological approaches that target single redox or ROS pathways (N-acetylcysteine, EGCG, Tocopherol) have shown mixed results in reducing the pathogenic progression of the muscular dystrophies (Scheuerbrandt, 2008), though combinatorial approaches may have better outcomes (Potgieter et al., 2011). In other diseases, genetic or pharmacological activation of Nrf2 has been shown to reduce oxidative and nitrosative stress and provide significant functional benefit (Thimmulappa et al., 2007; Sussan et al., 2009; Blake et al., 2010). Our results support a centralized role of Nrf2 deficiency in the pathogenesis of dysferlinopathy. We hypothesize that strategies targeting Nrf2 activation may ablate the core mechanistic hallmarks of dysferlinopathic progression (i.e., oxidative stress, inflammation, fibrosis, lipid accumulation). Further, as most muscular dystrophies share these mechanistic underpinnings, this approach, if successful, may have wide therapeutic applications.

### Conflict of interest statement

The authors declare that the research was conducted in the absence of any commercial or financial relationships that could be construed as a potential conflict of interest.
